# Red blood cell antibody screening in pregnancy

**DOI:** 10.4103/0973-6247.76002

**Published:** 2011-01

**Authors:** Shanthala A. M. Devi, Vanamala A. Alwar, S. Sitalakshmi, Karuna Rameshkumar, Rita Mhaskar

**Affiliations:** Department of Clinical Pathology, St Johns Medical College Hospital, Bangalore, India; 1 Department of Obstetrics and Gynaecology, St Johns Medical College Hospital, Bangalore, India

Sir,

Hemolytic disease of newborn (HDN) is defined as neonatal anemia and hyperbilirubinemia caused by an incompatibility between maternal and fetal red blood cells (RBCs).[1] In 98% cases it is caused due to ABO and Rh incompatibility and antibodies to other blood group antigens (Kell, c, E, C, Kidd, Duffy, M, and so on) are causative in remaining 2%.[2] More than 43 different RBC antigens have been reported to be associated with HDN.[3] Red cell antibody screening (RCAS) is a valuable tool in the detection of alloantibodies to other blood group systems (other than ABO and Rh) in the serum of patients during pregnancy or prior to transfusion. Red cell antibody identification (RCAI) should then be carried out on a larger panel of RBCs to precisely identify the antibody.

In a prospective study carried out on 624 antenatal cases, RCAS was done using a 3-cell panel from Diamed, Switzerland. RCAI was carried out on cases that were positive for RCAS. RCAS was positive in 9 out of 624 cases—1.4% (excluding the 3 cases who had autoantibodies). After RCAI these were identified as anti-D antibody (6 cases, 66%), anti-D with anti-C antibody (2 cases, 22%), and anti-M antibody (1 case, 11%).

The most common antibody identified remained anti-D. In 2 cases of Rh negative pregnancy, the RCAS was suggestive of anti-D. RCAI done, however, showed a combination of anti-D and anti-C. One case of anti-M was detected in a G_2_P_1_L_1_D_1_ lady. The first pregnancy was full-term normal delivery at home, however, the baby died after birth. The mother’s and baby’s blood group was O positive. RCAS done during second pregnancy was suggestive of anti-Duffy (Fy^a^) or anti-M antibody. RCAI done showed anti-M antibody with dosage effect. The second pregnancy was postdated with Intrauterine growth retardation (IUGR,) and Lower segment caesarean section(LSCS) was done for fetal distress. The baby had hyperbilirubinemia and was Direct Coombs test (DCT) positive requiring phototherapy.

Rh incompatibility continues to be a common cause for HDN. Patients with no prior history of sensitization can also develop anti-D as seen in 3 of our cases probably due to naturally occurring anti-Rh antibodies or antepartum hemorrhage. Despite the use anti-D immunization, 1%–2% of the cases are still sensitized. Anti-D immunization resulted in a favorable fetal outcome in the study. Anti-D complicated with anti-C presents with more severe HDN as seen in 1 patient who had a previous stillbirth and a hydrops baby despite receiving anti-D. Anti-C antibodies resulting in HDN requiring exchange transfusion have been reported.[4]

Anti-M as a cause of HDN is rare as they are usually complete cold antibodies (IgM). However, it can be IgG type resulting in HDN.[3] HDN due to anti-M antibody can be mild to severe with stillbirth and cases requiring exchange transfusion have been reported.[5] Anti-M causing blood group discrepancy and crossmatch incompatibility has been reported in the Indian literature.[6]

Antenatal detection of the non-anti-D causes of HDN requires RCAS. If RCAS is positive, the following steps are to be taken. RCAI should be done to identify the antibody. The spouse has to be screened for the presence of offending antigen and the pediatrician has to be alerted about delivery of a potentially sensitized infant. The blood bank should find a suitable antigen-negative donor for transfusion to baby and mother.
